# Knowledge of Bovine Tuberculosis, Cattle Husbandry and Dairy Practices amongst Pastoralists and Small-Scale Dairy Farmers in Cameroon

**DOI:** 10.1371/journal.pone.0146538

**Published:** 2016-01-08

**Authors:** Robert F. Kelly, Saidou M. Hamman, Kenton L. Morgan, Egbe F. Nkongho, Victor Ngu Ngwa, Vincent Tanya, Walters N. Andu, Melissa Sander, Lucy Ndip, Ian G. Handel, Stella Mazeri, Adrian Muwonge, Barend M. de. C. Bronsvoort

**Affiliations:** 1 The Roslin Institute, Royal (Dick) School of Veterinary Studies, University of Edinburgh, Easter Bush, Midlothian, United Kingdom; 2 Farm Animal Clinical Sciences, School of Veterinary Medicine, University of Glasgow, Glasgow, United Kingdom; 3 Institute of Agricultural Research for Development, Regional Centre of Wakwa, Ngaoundere, Cameroon; 4 Institute of Ageing and Chronic Disease and School of Veterinary Science, University of Liverpool, Leahurst Campus, Neston, Wirral, United Kingdom; 5 Microbiology and Parasitology Unit, Faculty of Allied Medical Science, University of Calabar, Nigeria; 6 School of Veterinary Medicine and Sciences, B.P. 454, University of Ngaoundere, Ngaoundere, Cameroon; 7 Cameroon Academy of Sciences, P.O. Box 1457, Yaoundé, Cameroon; 8 Ministry of Livestock, Fisheries and Animal Industries, NWR Regional Delegation, Bamenda, Cameroon; 9 Tuberculosis Reference Laboratory Bamenda, Hospital Roundabout, Bamenda, Cameroon; 10 Laboratory of Emerging Infectious Diseases, University of Buea, Buea, Cameroon; 11 The Royal (Dick) School of Veterinary Studies, University of Edinburgh, Easter Bush, Midlothian, United Kingdom; Cornell University, UNITED STATES

## Abstract

**Background:**

Control of bovine tuberculosis (bTB) and zoonotic tuberculosis (zTB) has relied upon surveillance and slaughter of infected cattle, milk pasteurisation and public health education. In Cameroon, like many other sub-Saharan African countries, there is limited understanding of current cattle husbandry or milk processing practices or livestock keepers awareness of bTB. This paper describes husbandry and milk processing practices within different Cameroonian cattle keeping communities and bTB awareness in comparison to other infectious diseases.

**Study design:**

A population based cross-sectional sample of herdsmen and a questionnaire were used to gather data from pastoralists and dairy farmers in the North West Region and Vina Division of Cameroon.

**Results:**

Pastoralists were predominately male Fulanis who had kept cattle for over a decade. Dairy farmers were non-Fulani and nearly half were female. Pastoralists went on transhumance with their cattle and came into contact with other herds and potential wildlife reservoirs of bTB. Dairy farmers housed their cattle and had little contact with other herds or wildlife. Pastoralists were aware of bTB and other infectious diseases such as foot-and-mouth disease and fasciolosis. These pastoralists were also able to identify clinical signs of these diseases. A similar proportion of dairy farmers were aware of bTB but fewer were aware of foot-and-mouth and fasciolosis. In general, dairy farmers were unable to identify any clinical signs for any of these diseases. Importantly most pastoralists and dairy farmers were unaware that bTB could be transmitted to people by consuming milk.

**Conclusions:**

Current cattle husbandry practices make the control of bTB in cattle challenging especially in mobile pastoralist herds. Routine test and slaughter control in dairy herds would be tractable but would have profound impact on dairy farmer livelihoods. Prevention of transmission in milk offers the best approach for human risk mitigation in Cameroon but requires strategies that improved risk awareness amongst producers and consumers.

## Introduction

*Mycobacterium bovis*, a member of the *Mycobacterium tuberculosis* complex (MTC), is primarily an infection of cattle but also various domestic and wild animal species [1]. The pathogen is the cause of bovine tuberculosis (bTB) and in chronically infected cattle can be associated with poor health and production [2,3]. Zoonotic transmission, from cattle to humans, is of great concern with approximately 3% of all human tuberculosis cases being caused by *M*. *bovis* [4,5]. It is generally believed that zoonotic transmission occurs through close contact with infected cattle or through consumption of untreated milk. Hence in many high-income countries the control of bTB in cattle is primarily aimed to protect human health rather than animal health [6–8]. The zoonotic risk of food borne transmission has been mitigated through public health initiatives such as meat inspection and processing milk by heating to a high temperature [9]. Increasing awareness of bTB, through education programs, has also been integral to zoonotic tuberculosis (zTB) control [8,10]. Bovine tuberculosis eradication programs have also relied upon test and slaughter of infected animals due to the chronic nature of bTB, lack of treatments and effective vaccines in livestock populations [11]. Yet in many low-income countries, where the majority of zTB cases occur, few control measures are present despite the high prevalence of bTB in cattle and the potential risk to public health [4,5,12]. In the face of advances in human TB treatment and control; TB is still prevalent worldwide with 3.3 million cases annually reported by the World Health Organisation (WHO) with 81% of cases occurring in low-income countries. With agriculture being the main form of income in rural Sub-Saharan communities, with many living in close contact with their livestock and consuming fresh milk products, it is unsurprising that zTB is of concern [5,6,13]. In addition to increased animal protein consumption, including fresh milk, in many sub-Saharan African countries. As the goal of the WHO, in its “END-TB” program, is to eliminate all forms of human tuberculosis by 2035 it is paramount zTB is not overlooked.

The prevalence of bovine tuberculosis in sub-Saharan extensively managed herds, such as in pastoral systems, is often high but with low within herd prevalence [14,15]. In high income countries, in the presence of control strategies, bTB prevalence between herds is low but where herds are infected the within herd prevalence is high. High within herd prevalence is often related to risk factors of intensive production systems involving housing cattle in close contact of one another [16–18]. Furthermore high bTB prevalence is also seen in intensive systems in sub-Saharan Africa [19]. Region-specific epidemiological data is often limited due to absence of cohesive disease surveillance systems [14,20,21]. It is likely that local practices will influence the variation in bTB prevalence and suggesting different local risk factors to *M*. *bovis* transmission[17]. Highlighting that understanding local cattle rearing systems is paramount prior to investigating bTB epidemiology. For example previous studies in Cameroon have reported bTB prevalence between 0.1–4.3% using lesion detection in abattoir based studies [22–24]. Higher prevalences have been reported using ante-mortem diagnostics (3.5–18.4%) such as the single comparative intradermal skin test and even higher using a serological assay high (37.2%)[25–27]. Furthermore the awareness of bTB and the extent of milk consumption within Cameroonian cattle keeping communities is poorly understood with only previous studies limited to butcher’s knowledge of bTB [22]. Yet active epidemiological surveillance in cattle is limited, resale of milk is unregulated and bTB education campaigns are absent. Cameroon is an important cattle-producing country within the Central and West Africa region, exporting cattle to adjacent countries such as Nigeria, Gabon and Congo in addition to supplying meat and milk for national consumption [28]. There are approximately six million cattle in Cameroon, mainly distributed over the mountainous North West Region (NWR), the Adamawa plateau and more northern Regions of Cameroon. Historically, Cameroonian cattle production has been undertaken by the Fulani ethnic group, a pastoral community spanning Central and West Africa [29,30]. Cattle keeping is core to Fulani culture, not only for meat and milk production, but importantly as financial capital. The importance of cattle is further highlighted by the more than 70 words for “cow” in the local Fulfulde language [31]. The Fulani graze mainly *Bos indicus* cattle breeds on extensive communal pastures and many herdsmen still practice transhumance (seasonal migration) in the dry season (November until April) along river valleys to find pasture. A sophisticated network of markets, trade routes and abattoirs join the value chain from the production areas to the large urban centres that are major consumers of livestock products. Over the past 10 years small-scale dairy farmer cooperatives have appeared; particularly in the NWR Region [32,33]. These dairy farmers tend to be from non-Fulani ethnic groups without a long tradition of cattle keeping and rear small numbers of *Bos taurus* cattle, mainly Holstein-Friesian type animals, semi-intensively in basic stalled housing. Milk is sold through their farmer cooperatives to peri/urban communities at local markets. The high cost of surveillance and limited veterinary/ public health infrastructure in Cameroon, like many sub-Saharan African countries, means that bTB control is challenging and likely to require a holistic approach [34,35]. Insight into bTB knowledge and milk processing practices in cattle keeping communities will improve our understanding of the socio-anthropological context and inform the scope and need for veterinary public health policies/strategies in Cameroon [36,37]. Further understanding of differences in cattle husbandry practices could aid identification of risk factors and potentially improve bTB control within Cameroon and adjacent countries.

This paper describes cattle husbandry, milk handling practices, knowledge and awareness of tuberculosis within a population based sample of herdsmen and dairy farmers from North West Region and Vina Division in Cameroon. Bovine tuberculosis knowledge and awareness will be compared to other important infectious diseases, such as foot-and-mouth diseases (FMD) and fasciolosis, to assess general infectious disease awareness. Reasons for variation in bTB awareness will be explored between pastoralists and dairy farmers.

## Materials and Methods

### Study sites

**Fig 1:**
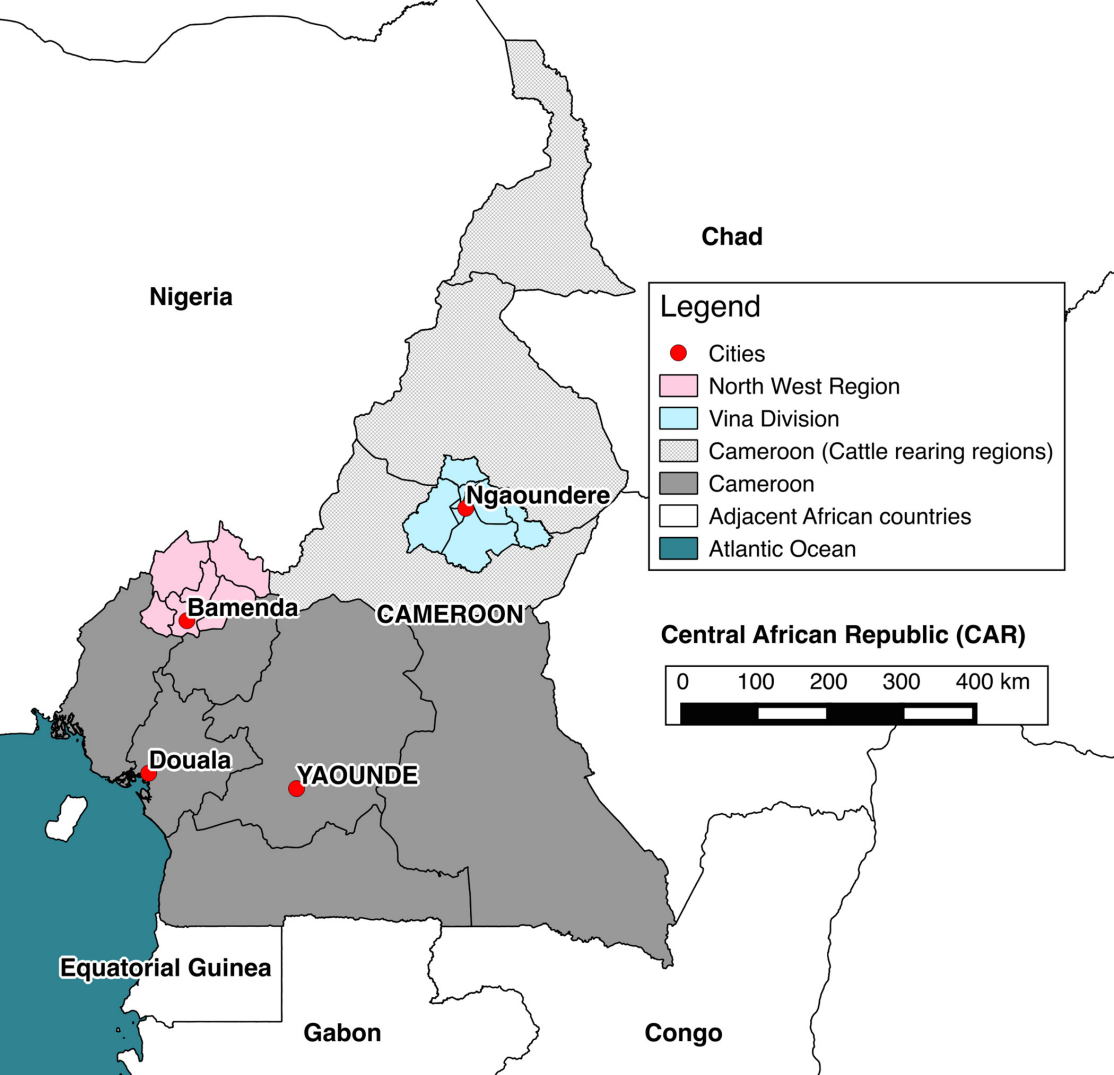
Map of Cameroon. The location of cattle rearing areas (light grey), study sites (pink and blue) and major cities (red).

The study sites were the North West Region (NWR) and Vina Division (VD) of the Adamawa Region of Cameroon. Both are of similar geographical size of ~17,000km^2^ (Fig 1). The NWR is an anglophone region situated in fertile mountainous highlands, 500-3000m above sea level. Bamenda, the capital, is Cameroon's third largest city. The Region is densely populated (1,804,695 people) and an estimated 506,548 cattle are grazed there [38,39]. The VD is part of the fertile Adamawa Region’s savannah plateau. The regional capital is Ngaoundere and the mainly francophone population of the VD (317,888 people) is much smaller than that of the NWR. The cattle population is also smaller with an estimated 176,257 head [40].

Veterinary services are predominately provided by the government through the Ministry of Livestock, Fisheries and Industrial Agriculture /Ministere de l’Elevage des Peches et Industries Animales (MINEPIA), with local veterinary technicians stationed at Zootechnical and Veterinary Sanitary Control Centres (ZVSCC) distributed across the country [41]. Their responsibilities include registration of local livestock keepers, disease control mainly through annual vaccination campaigns, meat inspection and regulation of livestock markets and animal movements.

### Study design

Please note that we include brief mention of some details related to animal sampling for completeness so the reader can appreciate the context in which the herdsmen and farmer surveys were conducted but we will not present animal level data here as this analysis will focus only on the herdsmen and farmer knowledge and attitudes. Two cross sectional surveys were conducted between January–May 2013 in the NWR and September-November 2013 in the VD. The first was of pastoralists whose herds were listed in the Ministry of Livestock, Fisheries and Animal Industries vaccination records at 81 ZVSCCs in the NWR and 31 ZVSCCs in the VD in 2012. A total of 5,053 pastoralist herds in the NWR and 1,927 in the VD, with a range of 1–215 cattle per herd were included in the sampling frame. The list of herds in each site was stratified by administrative area; seven Divisions in the NWR and eight sub-Divisions within the VD and a random sample of herds was taken from each site proportional to the total number herds listed in each strata. This survey was part of a larger study of bTB and liver fluke and the sample size was based on a clustered random sample of cattle assuming a cattle level prevalence of ~10%, a within herd variance of 0.15 and between herd variance of 0.01, an average herd size of 70, a relative cost of 12:1 for herd:cattle and relative error of ±15% (Survey Toolbox; AusVet) [42]. This gave a target sample size of 15 cattle per herd and 88 herds under the simplifying assumption of perfect test performance. To allow for potential losses or drop out and to have balanced samples from the 2 sites, we aimed for 50 herds each of the NWR and VD and therefore herdsmen to be interviewed. Hence note that within the NWR or VD the estimates are unbiased but overall estimates require weighting to adjust for the different sampling intensities in the NWR and VD.

The second survey was of the small-scale dairy farmers who were all registered with Ministry of Livestock, Fisheries and Animal Industries and the address list for 2012 was obtained from their NWR office in Bamenda. Dairy co-operatives were established as part of a non-governmental organisation (NGO) initiative in the 1990s to improve milk production in the area. Donated Holstein-Friesian cattle from Ireland and Kenya were imported and given to families to be reared in zero grazing systems [33]. Calves born from these original cattle were then passed on to other members joining the cooperatives. There were 229 dairy farmers, grouped into 13 cooperatives with 3 to 52 farmers per cooperative. The cooperatives were categorised geographically into 4 groups. Three were spatially clustered with three cooperatives in each group. The fourth group consisted of four widely dispersed cooperatives and was not sampled for logistical reasons. Thus 164 dairy farmers were included in the sample frame. A stratified random sample of dairy farmers was selected proportional to the number of dairy farmers in each group. Again the survey was part of a wider study of bTB and fasciolosis and sampling cattle but based on the assumption of perfect test performance, a prevalence of ~6% in adult cattle (Nkongho et al, in press) and each dairy farmer having two adult cows resulting in a sample size of 46 dairy farmers.

Selected pastoralists and dairy farmers were contacted by phone or in person by the head of the local ZVSCC, and asked if they were prepared to participate in the study. Individuals were replaced by resampling if they declined, had died, moved out of the region, or were located more than three hours walk from a point that could be accessed by off-road vehicle, motorbike or on horseback.

### Data collection

Pastoralist herdsmen were visited either at their homestead or at a convenient location in the vicinity where the cattle could be examined. Dairy herds were visited at the homestead. The translator/research assistant (SH) explained the project in either Fulfulde, Pidgin, English or French and the herdsman or farmer was asked to give verbal consent to participating in the study. It was made clear that this included completing a questionnaire about the management of their cattle and their knowledge of bTB, allowing the single cervical intradermal skin test (SCITT) to be conducted and a blood sample to be taken from their cattle for further diagnostic tests such as the bTB Υ-interferon assay (Bovigam®) [3,43].

A structured questionnaire (S1 File) was administered by interview (SH) in the respondents preferred language. The questionnaire was developed through discussions with pastoralists, veterinary professionals and researchers. The questionnaire was pretested and modified prior to final use. The questionnaire took 20–30 minutes to administer. Questions asked focused on herdsman background, dairy practices routine herd practices, herd reproduction, grazing/ housing, transhumance, cattle trade, and infectious diseases. Specifically knowledge and awareness of bTB was investigated but also FMD and fasciolosis as comparisons. Local names were used where appropriate such “Soharu”, “Balki” and “Njobu” were used in Fulfulde and “Tuberculose bovine”, “Douve du foie” and “Fièvre aphteuse” in French. Cameroonian Pidgin names for these diseases are the same as in English. Awareness of an infectious disease was defined as “the participant recognising the name of the disease”. If pastoralists or dairy farmers were not aware of a particular infectious disease no more questions were asked relating to that disease. GPS location (Garmin eTrex® Venture) was also recorded. The questionnaire data were initially recorded in paper format and then transferred to a relational Access database (Microsoft Access®).

### Statistical analysis

Samples sizes relate to the number of pastoralists and dairy farmers sampled. Statistical analyses were performed using packages and functions in R Studio 0.98® [44]. For pastoralists the study design was incorporated using the *svydesign* function in the *survey* package [45]. Descriptive statistics were estimated using *svymean*, *confint* and *svyby* functions to account for the design effects. Graphics were produced using the *ggplot2* package [46]. Maps were drawn using QGIS 2.2® [47] and shape files obtained from the open access GADM database of Global Administrative Areas (www.gadm.org). The multivariable mixed logistic regression models were developed using the R package *stats* and *glm* functions [48]. The main outcome variable was the dichotomous answer to the question “Are you aware of a disease called “Bovine Tuberculosis?”. Similar questions were asked for fasciolosis and FMD. Explanatory variables were categorized as appropriate eg. “Location” was catagorized as strata (NWR and VD) for pastoralists and cooperative group for dairy farmers. A backwards stepwise approach was used to find the best fitting model to describe the dataset [49]. Model selection was based on the Akaike information criterion (AIC) and the best model was selected using the lowest AIC. Final model selection was verified by computing AICc and ΔAIC using the R package *AICcmodavg* and *modavg* functions [50]. The p value, odds ratio with 95% CI for explanatory variables were also calculated.

### Ethics statement

The study design and sampling methodology was reviewed and approved by the University of Edinburgh Ethics Committee, UK (ERC No: OS02-13) and by the Institute of Research and Development (IRAD), Cameroon. IRAD gave permission to conduct the fieldwork and issued fieldwork permits. The research did not involve endangered or protected species and no further approvals were necessary to conduct fieldwork. All participants gave informed verbal consent to participate and were aware they could opt out at any stage. Verbal consent was deemed appropriate for the variety of dialects spoken, variable literacy amongst participants and due to the remote outdoor fieldwork environment [41,51]. Information to be provided to participants, for informed verbal consent, was communicated to the interviewer (SH) in a written document. Additional training was provided to the interviewer regarding the consent procedure and interview process. Furthermore the interviewer was experienced in conducting questionnaires in similar studies and spoke the various local dialects of study participants [41]. Verbal consent was recorded on a cover sheet to the questionnaire by the interviewer and refusals were recorded in separate document along with reasons for refusal.

## Results

In total 100 pastoralists were interviewed; 50 in the NWR and 50 in the VD. Of the selected herdsmen 23 were unavailable and these were replaced by randomly resampling from within the same ZVSCC list. Reasons for replacement included moving away from the study area (n = 4); had <10 cattle (n = 4), no longer kept cattle (n = 3); logistical issues (n = 6); herdsman name selected not known (n = 3); declined to participate at interview stage (n = 2) and the herdsman had died and their herd dispersed (n = 1). All 46 selected dairy farmers participated and none were replaced.

### Participants, cattle and husbandry practices

Overall, 97.8% (CI: 86.4–99.7%) of interviewed pastoralists in the NWR and 100% (CI: 92.9–100%) in the VD were male. In contrast 43.5% (CI: 29.0–58.0%) of dairy farmers were female. There were differences in their formal schooling; 63.2% (CI: 50.0–74.7%) of NWR pastoralists and 74.0% (CI: 60.6–84.2%) of those in the VD had no formal schooling whereas all dairy farmers had some form of schooling, 76.1% (CI: 63.3–88.5%) at “primary school” level. The majority of pastoralists identified themselves as members of a Fulani ethnic group. In the NWR; 89.4% (CI: 77.4–95.4%) were "Mbororo" and only 2.0% (CI: 0.2–12.7) “Fulbe’; while in the VD 66.1% (CI: 53.0–77.1%) considered themselves as "Fulbe" and 17.6% (CI: 9.9–29.5) as “Mbororo”. The remainder were non-Fulani ethnic groups. None of the dairy farmers considered themselves to be from either Fulani group. Pastoralists in the NWR had worked with cattle for longer (26.5 years, CI: 22.4–30.5 years) than those in the VD (17.7 years, CI: 13.7–21.4). This was considerably longer than dairy farmers (5.5 years, CI: 4.0–6.9). Yet the mean ages of pastoralists in the NWR (41.0 years, CI: 37.0–44.9), VD (39.2 years, CI: 35.3–43.4) and dairy farmers were similar (45.8 years, CI: 42.4–49.3).

Reported pastoral herd sizes were larger in the NWR (50, CI: 45–55) than in the VD (38, CI: 34–43). Both were much larger than dairy herds (3, CI: 2–3). All pastoral cattle were *Bos indicus* or *Bos inducus/ Bos taurus* cross-breeds. Mixed breed (63.9%, CI: 54.6–72.2%) and red (16.1%, CI: 10.1–24.7%) and white Fulani (20.0%, CI: 12.9–29.8%) were mainly kept by NWR. In the VD Gudali cattle (83.5%, CI: 78.1–88.8%) were the most common breed. Almost all dairy farmers kept *Bos taurus* Holstein-Friesian cattle (98.3%, CI: 95.1–100%).

All pastoralists (CI: 92.9–100%), in the NWR and VD managed their cattle extensively and 97.9% (CI: 86.6–99.7%) used streams as a source of drinking water. Almost all the pastoralists in both study sites contacted other herds during grazing (94.0%, CI:88.8–99.3%) and 67.0% (CI: 58.2–75.9%) had contact during watering; contacting one to 15 herds on a daily basis. Keeping cattle in fenced enclosures overnight was more common in the NWR (54.7%, CI: 43.1–66.3%) than in the VD (17.1%, CI: 8.8–25.3%). In contrast, 97.8% (CI: 93.6–100%) of dairy farmers housed their cattle and all (CI: 92.3–100%) used water troughs. Consequently, only 8.7% (CI: 0.5–16.9%) of dairy herds had contact with other herds.

There were regional and ethnic differences in the practice of transhumance. A greater proportion of NWR pastoralists (43.8% CI: 31.4–57.1%) undertook transhumance compared to with those in the VD (6.2% CI: 2.0–17.7%). Furthermore, across both pastoralist communities, 44.0% (CI: 30.1–58.0%) of Mbororo herdsmen went on transhumance compared with 10.7% (CI: 0–25.2%) of Fulbe. During transhumance, all herds came into contact with one to 15 herds on a daily basis.

When asked about contact with potential wildlife reservoir hosts, antelope were the most frequently contacted species. During normal grazing fewer herds in the NWR (49.8%, CI: 36.0–63.6%), reported antelope contact than in the VD (76.4%, CI:65.4–84.7%). During transhumance 80.9%, (CI: 64.5–97.3%) n = 25) reported antelope contact. Contact with buffalo was only reported during transhumance; it involved 25.4% herds (CI: 8.9–41.8%). Warthog contact was reported by 11.9% of herdsmen in NWR (CI: 5.4–24.0%) and 38.2% in the VD (CI:27.2–50.6%) when grazing and by 32.9% (CI:15.1–50.7%) during transhumance. All dairy farmers reported that none of the cattle came into contact with wildlife.

Natural service was used for breeding by all pastoralists (CI: 92.9–100%) and 89.1% (CI: 80.0–98.2%) of dairy farmers. In addition, artificial insemination (AI), using *Bos taurus* semen, was used by 10.2% (CI: 4.4–22.0%) of NWR pastoralists, 2.0% (CI: 0.0–5.8%) of VD pastoralists and 8.0% (CI: 1.7–14.4%) dairy farmers. Cattle selling was reported by 93.8% (CI: 83.2–97.9%) and 83.9% (CI: 71.3–91.6%) of pastoralists, in the NWR and VD respectively. A smaller proportion of pastoralists reported buying cattle in both the NWR (41.8%, CI: 30.0–54.7%) and VD (49.7%, CI: 36.4–62.9%). Most pastoralists in the NWR (83.4%, CI: 70.0–91.5%, n = 42) and VD (87.8%, CI: 75.4–94.4%, n = 44) traded at markets. Comparatively few dairy farmers sold cattle (37.0%, CI: 22.9–51.1%), purchased cattle (8.7%, CI: 0.4–16.9%) or traded at markets (11.8%, CI: 0.0–27.6%,).

When asked about treatments, anthelmintics (Albendazole or Ivermectin) use was reported by 100% (CI: 92.3–100%) of dairy farmers, 93.9% (CI: 82.6–98.1%) of pastoralists in the NWR and 84.2% (CI: 71.2–92.0) of those in the VD. Trypanosomiasis treatment was used by 77.7% (CI: 65.9–86.2%) of pastoralists in the VD compared with fewer 41.9% (CI: 29.1–56.0%) in the NWR. No dairy farmers treated for trypanosomiasis (CI: 0.0–8.6%).

Of the other susceptible species kept, goats were reported by about a third of pastoralists and dairy farmers; NWR (29.9%,CI: 19.2–43.3), VD (27.2%,CI:16.7–27.2), Dairy (30.4%,CI:20.9–48.7) and sheep by 45.2% (CI: 33.7–57.1), 28.8% (CI: 18.7–41.6) and 23.9% (CI: 11.5–36.4) of herdsmen respectively. Poultry were the most common species kept and were reported by 75.4% (CI: 61.2–85.7%), pastoralists in the NWR 65.4%, (CI: 51.1–77.4%) in VD and by 63.0%, (CI: 48.9–77.1%). dairy farmers. Horses were kept by pastoralists in the NWR (39.9%, CI: 28.2–53.0%) but were rarely kept by those in the VD (2.0%, CI: 0.3–12.4%) or by dairy farmers (2.2%, CI: 0.0–6.4%).

### Knowledge of bovine tuberculosis compared to other infectious diseases

**Fig 2:**
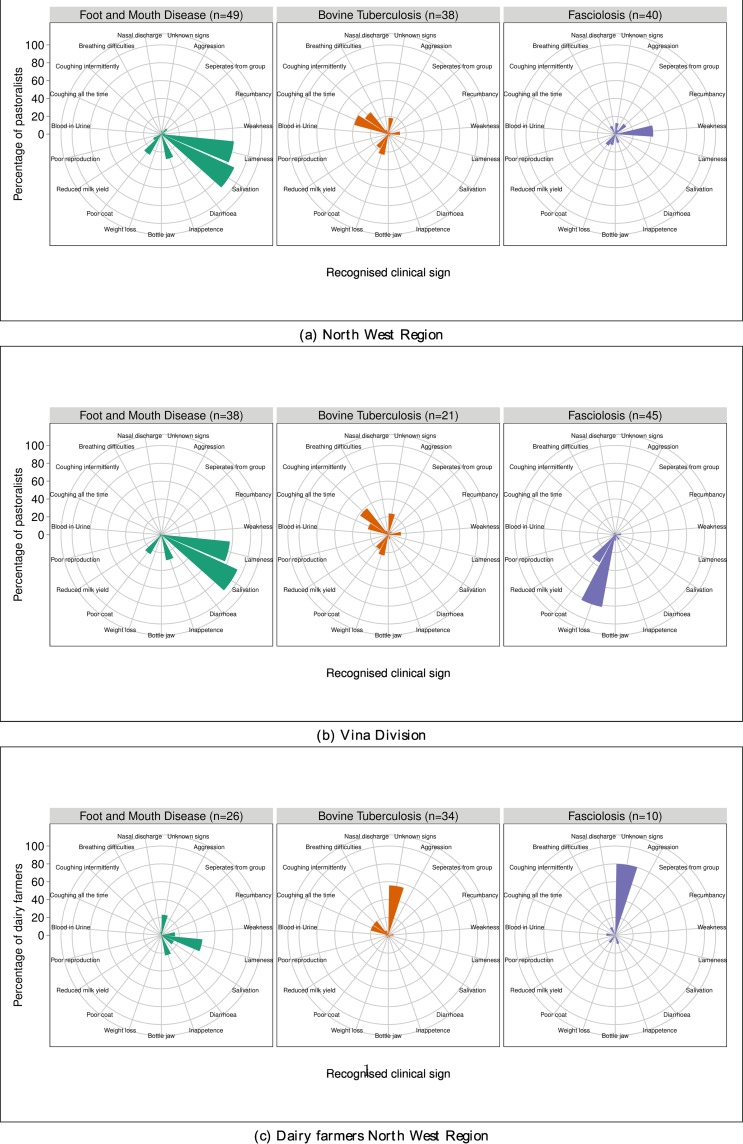
Frequency of clinical signs identified for bovine tuberculosis and fasciolosis. Y-axis intervals are for every 20% of cattle keepers “aware” of the disease.

More dairy farmers (73.9%, CI: 61.1–86.7%) and NWR pastoralists (76.6%, CI: 63.4–86.1%) were “aware” of bTB than VD pastoralists (40.8%, CI: 30.1–52.5%). Nearly a quarter of herdsmen in the NWR reported cattle having died from tuberculosis or been informed about it from slaughter cases compared to <10% of those from the VD (Table 1). In contrast 4.3% (CI: 0.0–10.3%, n = 34) of dairy farmers had previously had a bTB SCITT conducted within their herd and an animal reported positive. The proportion of pastoralists that could not identify clinical signs for bTB was 18.4% in the NWR (CI: 5.9–30.9%, n = 38) and 23.8% (CI: 5.1–42.5%, n = 21) in the VD. But over half of dairy farmers (55.9%, CI: 38.9–72.8%, n = 34), who were aware of bTB, could not identify any clinical signs for bTB. Pastoralists, who were aware of bTB, identified coughing, weight loss, poor coat and weakness as signs of bTB and the pattern for the NWR and VD are almost identical (Fig 2).

**Table 1:** Proportion of herds reported to have had various infectious diseases based on the subset of pastoralist and dairy farmers who reported being aware of the given infectious disease.

	North West Region Pastoralists (95% CI)	Vina Division Pastoralists (95% CI)	North West Region Dairy Farmers (95% CI)
**BOVINE TUBERCULOSIS (bTB)**	(n = 38)	(n = 21)	(n = 34)
**Have you been informed of any cattle sold or slaughtered have bTB?**			
*Yes*	23.5% (13.1–38.5%)	8.5% (2.1–27.8%)	0.0% (0.0–7.7%)
**FASCIOLOSIS**	(n = 40)	(n = 45)	(n = 10)
**Have any of the cattle presented been sick from fasciolosis?**			
*Yes*	47.5% (32.3–63.3%)	34.4% (22.4–48.9%)	0.0% (0.0–7.7%)
**Have any of your cattle died from fasciolosis?**			
*Yes*	35.2% (21.6–51.8%)	10.7% (4.5–23.4%)	0.0% (0.0–7.7%)
**Have you been informed of any cattle sold or slaughtered have fasciolosis?**			
*Yes*	15.2% (6.9–30.1%)	28.3% (17.1–43.0%)	0.0% (0.0–7.7%)
**FOOT AND MOUTH DISEASE (FMD)**	(n = 49)	(n = 48)	(n = 26)
**Have any of the cattle presented been sick from FMD?**			
*Yes*	60.1% (46.6–72.2%)	76.4% (62.6–86.2%)	7.7% (0.0–18.1%)
**Have any of your cattle died from FMD?**			
*Yes*	32.9% (21.5–46.8%)	16.8% (8.8–29.7%)	3.8% (0.0–11.4%)

More pastoralists in the NWR (80.1%, CI: 68.1–88.3%) and VD (89.9%, CI: 78.4–95.6%) were aware of fasciolosis than dairy farmers (21.7%, CI: 9.7–33.8%). More pastoralists in the VD reported fasciolosis in their cattle than pastoralists in the NWR and dairy farmers (Table 1). In the NWR weakness was the most frequently reported clinical sign for fasciolosis (42.5%, CI: 27.0–58.0%, n = 40). Other clinical signs reported, by NWR pastoralists, were poor coat, bottle jaw, inappetence, separating from the group, nasal discharge and breathing difficulties. Weight loss (82.2%, CI: 70.9–93.5%, n = 45) was the most frequently reported by VD pastoralists along with poor coat, bottle jaw, inappetence and weakness (Fig 2). Only 12.5%, (CI: 2.1–22.9%, n = 40) of pastoralists in the NWR and 2.2% (CI 0.0–6.6%, n = 45) in the VD were unable to identify any clinical signs for fasciolosis when compared to the majority of dairy farmers (80.0%, CI: 53.9–100%, n = 10).

In comparison more pastoralists in the NWR (97.9%, CI: 86.6–99.7%) and VD (96.0, CI: 85.4–99.0%) were “aware” of FMD than dairy farmers (56.5%, CI: 42.0–71.0%). All pastoralists could identify at least one clinical sign of FMD whereas 23.1% (CI: 6.5–39.6%, n = 26) of the dairy farmers were unable to identify any clinical signs of FMD. Pastoralists frequently reported salivation (NWR: 89.8%, CI: 81.2–98.4%, n = 49; VD: 93.8%, CI: 86.8–100%, n = 48)) and lameness (NWR: 81.6%, CI: 70.7–92.6%, n = 49; VD:77.1%, CI: 65.1–89.1%, n = 48) as clinical signs of FMD along with inappetence, poor coat, reduced milk yield and separation from the group (Fig 2).

### Dairy practices and zoonotic tuberculosis

Milk was consumed by the majority of dairy farmers’ (87.0%, CI: 77.1–96.8%) and pastoralists’ families in both the NWR (87.7%, CI: 77.6–93.6%) and VD (96.0%, CI: 85.4–99.0%). There were differences in processing and production of milk between dairy farmers and pastoralists. Dairy farmers only processed milk by heating whereas pastoralists processed it by souring or heating. All dairy farmers heated milk for their families (CI: 91.2–100%, n = 40). Processing milk for pastoralist’s families was more common in the VD (95.8%, CI: 84.8–98.9%, n = 48) than the NWR (74.0%, CI: 61.7–83.4%, n = 44). Furthermore pastoralists in the VD heated (87.5%, CI: 75.5–94.2%) and soured (87.1%, CI: 75.0–93.8%, n = 48) milk more frequently than in the NWR (60.8% (CI: 47.1–73.0%, n = 44) and 55.4% (CI: 43.8–66.5%, n = 44) respectively).

The majority of dairy farmers were producing milk for non-family members (87.0%; CI: 77.1–96.8%) compared to only 42.2% (CI: 29.4–56.1%) of pastoralists in the NWR and 16.5% (CI: 10.0–26.1%) in the VD. However, interestingly only 27.5%, (CI: 13.5–41.5%, n = 40) of dairy farmers processed milk for sale compared to 46.9% (CI: 27.6–76.1%) in the NWR and 9.0%, (CI: 1.3–41.0%, n = 9) in the VD. For those that did process for non-family members, dairy farmers reported heating and in the NWR 41.8% (CI: 23.5–62.7%, n = 21) and VD 9.0% (CI: 1.3–41.0%, n = 9) of pastoralists reported souring milk for non-family members. Dairy farmers and pastoralists produced butter and yoghurt. Fewer dairy farmers (8.7%, CI: 0.5–16.9%) produced butter than pastoralists in the NWR (65.7%, CI: 57.7–76.7%) and VD (75.4%, CI: 62.6–84.9%). Yoghurt was produced by 61.4%, (CI: 50.0–71.7%) of pastoralists in the NWR, 83.6%, (CI: 70.8–91.4%) in the VD and 45.7%, (CI: 31.1–60.2%) of dairy farmers. The median time for milk being soured was one day for pastoralists in the NWR (IQR: 1–2 days) and VD (IQR: 1 day).

Awareness of disease transmission via milk was reported by about a quarter of pastoralists in the NWR (28.3%, CI: 17.3–42.6%) and VD (26.1%, CI: 15.6–40.3%) and about half of the dairy farmers (56.5%, CI: 42.0–71.0%). However, only a small proportion of NWR and VD pastoralists understood that bTB could be transmitted to people through milk, 9.7% (CI: 4.1–21.1%) and 2.0% (CI: 0.3–12.3%) respectively compared with 21.7% (9.7–33.8%) of dairy farmers. Note all descriptive results are also displayed in tabular format (S2 File).

### Multivariable regression models for disease awareness

Multivariable models were developed to identify factors associated with the awareness of herdsmen and farmers of the three difference diseases discussed in the questionnaire. The pastoralist and dairy farmers were modelled separately as the husbandry systems were so different. Backward stepwise selection of final models is demonstrated in Table 2. Table 3 shows the final model for bTB awareness and interestingly it suggests that pastoralists in the VD are much less likely to be aware of bTB than those in the NWR. Awareness of fasciolosis and FMD in pastoralists was not associated with Region. For the dairy farmers awareness of bTB, fasciolosis and FMD were all associated with being male (Table 3). There was no association with schooling, ethnic group, age, years kept cattle, job type, milk-processing practices or trading cattle in either pastoralists or small-scale dairy farmers.

**Table 2:** Comparison of mixed-effects logistic regression risk factor models for disease awareness. **(a)** “Are you aware of a disease in cattle called bTB?” (BTBYNU) in pastoralists (n = 100), **(b)** “Are you aware of a disease in cattle called bTB?” (BTBYNU) in dairy farmers (n = 46), **(c)** “Are you aware of a disease in cattle called fasciolosis?” (LFLYNU) in dairy farmers (n = 46,) and **(d)** “Are you aware of a disease in cattle called foot and mouth disease?” (FMDYNU) in dairy farmers (n = 46). Explanatory variables included are OWNSEX (Gender), OWNEDU (Education level), OWNETH (Ethnic group), OWNAGE (Age), OWNCTY (Years worked with cattle), WHOYOU (Job title for pastoralist only), FAMTRT (Do you treat milk?), BUYSAL (Do you trade cattle? For pastoralist only), strata1 (Pastoralist study site) and SUBDIV (Dairy farmer cooperative group). Selected model = *

**(a) Pastoralist—**Are you aware of a disease in cattle called bTB? **(BTBYNU) (n = 100)**				
**Model**	**K**	**AIC**	**AICc**	**ΔAIC**
BTBYNU~1 + OWNSEX + OWNEDU + OWNETH + OWNAGE + OWNCTY + WHOYOU + FAMTRT + BUYSAL + strata1	18	148.29	156.84	29.58
BTBYNU~1 + OWNSEX + OWNEDU + OWNETH + OWNCTY + WHOYOU + FAMTRT + BUYSAL + strata1	17	146.43	153.90	26.64
BTBYNU~1 + OWNSEX + OWNEDU + OWNETH + OWNCTY + WHOYOU + BUYSAL + strata1	15	142.67	148.38	21.12
BTBYNU~1 + OWNSEX + OWNEDU + OWNETH + WHOYOU + BUYSAL + strata1	13	139.26	143.49	16.23
BTBYNU~1 + OWNSEX + OWNEDU + OWNETH + WHOYOU + strata1	11	135.79	138.79	11.53
BTBYNU~1 + OWNSEX + OWNETH + WHOYOU + strata1	8	132.43	134.01	6.75
BTBYNU~1 + OWNSEX + OWNETH + strata1	6	130.37	131.27	4.01
BTBYNU~1 + OWNSEX + strata1	3	128.58	128.83	1.57
BTBYNU~1 + OWNSEX	2	138.31	138.43	11.17
BTBYNU~1 + strata1*	2	127.14	12.26	0.00
**(b) Dairy farmer—**Are you aware of a disease in cattle called bTB? **(BTBYNU) (n = 46)**				
Model	**K**	**AIC**	**AICc**	**ΔAIC**
BTBYNU~1 + OWNSEX + OWNEDU + OWNAGE + OWNCTY + FAMTRT + SUBDIV	11	57.90	65.90	16.29
BTBYNU~1 + OWNSEX + OWNEDU + OWNCTY + FAMTRT + SUBDIV	9	55.46	60.46	10.85
BTBYNU~1 + OWNSEX + OWNCTY + FAMTRT + SUBDIV	6	52.64	54.79	5.18
BTBYNU~1 + OWNSEX + OWNCTY + FAMTRT	4	50.17	51.15	2.54
BTBYNU~1 + OWNSEX + FAMTRT*	3	49.04	49.61	0.00
BTBYNU~1 + FAMTRT	2	54.97	55.25	5.64
BTBYNU~1 + OWNSEX	2	50.12	50.40	0.79
**(c) Dairy farmer—**Are you aware of a disease in cattle called fasciolosis? **(LFLYNU) (n = 46)**				
Model	**K**	**AIC**	**AICc**	**ΔAIC**
LFLYNU~1 + OWNSEX + OWNEDU + OWNAGE + SUBDIV + FAMTRT + OWNCTY	11	44.44	52.44	12.63
LFLYNU~1 + OWNSEX + OWNEDU + SUBDIV + FAMTRT + OWNCTY	9	41.77	46.77	6.96
LFLYNU~1 + OWNSEX + OWNEDU + SUBDIV + OWNCTY	8	39.95	43.84	4.03
LFLYNU~1 + OWNSEX + SUBDIV + OWNCTY	5	38.31	41.90	2.08
LFLYNU~1 + OWNSEX + OWNCTY*	3	37.75	39.81	0.00
LFLYNU~1 + OWNSEX	2	42.79	40.76	0.95
LFLYNU~1 + OWNCTY	2	45.48	50.44	10.53
**(d) Dairy farmer—**Are you aware of a disease in cattle called foot and mouth disease? **(FMDYNU) (n = 46)**				
Model	**K**	**AIC**	**AICc**	**ΔAIC**
FMDYNU~1 + OWNSEX + OWNEDU + OWNAGE + OWNCTY + FAMTRT + SUBDIV	11	69.13	77.13	17.15
FMDYNU~1 + OWNSEX + OWNAGE + OWNCTY + FAMTRT + SUBDIV	8	64.22	68.73	8.75
FMDYNU~1 + OWNSEX + OWNAGE + OWNCTY + FAMTRT	6	61.69	63.89	3.91
FMDYNU~1 + OWNSEX + OWNCTY + FAMTRT	4	61.35	62.32	2.34
FMDYNU~1 + OWNSEX + OWNCTY*	3	59.41	59.98	0.00
FMDYNU~1 + OWNSEX	2	61.31	60.47	0.49
FMDYNU~1 + OWNCTY	2	60.19	61.59	2.11

**Table 3:** Final mixed-effects logistic regression risk factor models for disease awareness. **(a)** “Are you aware of a disease in cattle called bTB?” (BTBYNU) in pastoralists (n = 100), **(b)** “Are you aware of a disease in cattle called bTB?” (BTBYNU) in dairy farmers (n = 46), **(c)** “Are you aware of a disease in cattle called fasciolosis?” (LFLYNU) in dairy farmers (n = 46,) and **(d)** “Are you aware of a disease in cattle called foot and mouth disease?” (FMDYNU) in dairy farmers (n = 46). Explanatory variables included are OWNSEX (Gender) and strata1 (Pastoralist study site).

**(a) Pastoralist—**Are you aware of a disease in cattle called bTB? **(BTBYNU) (n = 100)**				
Final model: BTBYNU~1 + strata1				
**Variables**	**Levels**	**Odds ratio**	**95% CI**	**p value**
strata1	North West Region	1		
	Vina Division	0.23	0.09–0.53	<0.01
**(b) Dairy farmer—**Are you aware of a disease in cattle called bTB? **(BTBYNU) (n = 46)**				
Final model: BTBYNU~1 + OWNSEX				
**Variables**	**Levels**	**Odds ratio**	**95% CI**	**p value**
OWNSEX	Female	1		
	Male	8.63	1.86–63.21	0.01
FAMTRT	No	1		
	Yes	6.46	0.81–68.16	0.09
**(c) Dairy farmer—**Are you aware of a disease in cattle called fasciolosis? **(LFLYNU) (n = 46)**				
Final model: LFLYNU~1 + OWNSEX				
**Variables**	**Levels**	**Odds ratio**	**95% CI**	**p value**
OWNSEX	Female	1		
	Male	21.91	3.05–461.65	<0.01
OWNCTY	> = 5 years	1		
	<5 years	0.12	0.01–0.65	0.04
**(d) Dairy farmer—**Are you aware of a disease in cattle called foot and mouth disease? **(FMDYNU) (n = 46)**				
Final model: FMDYNU~1 + OWNSEX				
**Variables**	**Levels**	**Odds ratio**	**95% CI**	**p value**
OWNSEX	Female	1		
	Male	3.74	1.01–14.77	0.05
OWNCTY	> = 5 years	1		
	<5 years	3.27	0.81–14.75	0.10

## Discussion

This study describes the knowledge of bovine tuberculosis, cattle management and milk processing practices amongst pastoralist and small-scale dairy farmers in two Regions of Cameroon. Such studies are important because as WHO moves towards its aim of eradicating tuberculosis by 2050 [52]; the relative importance of zoonotic tuberculosis (zTB) caused by *M*. *bovis* may increase particularly in sub-Saharan Africa with its developing dairy industries [53]. In this Region future interventions preventing the zoonotic spread of tuberculosis are likely to be aimed at preventing transmission in milk and reducing the herd prevalence. Milk producers and cattle herds will be the targets of control strategies. Hence improving our understanding of livestock keepers knowledge of bTB, cattle management and milk processing practices which may favour *M*. *bovis* transmission are important.

In common with many countries in sub-Saharan Africa, Cameroon has a small-scale dairy industry based on imported *Bos taurus* and their cross-breeds [32]. This study indicates that the herd structure, contact and movement patterns of these herds and the awareness of bTB amongst dairy farmers were very different from the traditional *Bos indicus* keeping pastoralists. Small-scale dairy farmers had small numbers of Holstein-Friesian cows with little reported contact with other herds or wildlife. Although the control of tuberculosis in this population may appear tractable, e.g. by closing herds, using artificial insemination and test and slaughter, there are a number of caveats to this. These herds were set up with a limited number of imported animals from Ireland and Kenya. Both of these countries report endemic bTB and in the absence of a perfect test, importation of bTB as well as cattle cannot be discounted. The occurrence of pseudo-vertical transmission means that one infected herd may have given rise to a number of secondarily infected herds [16]. For example as there is a reliance on using shared bulls as the majority of dairy herds use natural service and an infected bull could potentially infect numerous herds. Furthermore although dairy herd sizes are small; test and slaughter policies would have a major impact on the livelihoods of these farmers and sustainable public health measures should be considered in this context.

In comparison the control of tuberculosis amongst the large pastoralist herds, by movement and contact restrictions, presents a much greater challenge and is probably impossible in the absence of an effective vaccine. These herds are relatively large and had daily contact with other herds and wildlife especially antelopes and warthogs. Additionally the practice of transhumance dramatically increased the number and range of contacts with other herds and wildlife. The importance of wildlife contact is indicated by studies in Zambia where the prevalence of bTB in cattle was proportional to its prevalence in wildlife [54]. Wildlife in South Africa have been shown to carry the same spoligotypes of *M*. *bovis* as cattle but transmission ecology is unclear[55]. Species of antelope in Cameroon include eland (*Taurotragus spp*), roan antelope (*Hippotragus spp*), korrigum (*Damaliscus spp*), kob (*Kobus spp*) and duiker (*Cephalophus spp)* [56,57]. These species of antelope are different to those in South Africa and interaction between cattle and wildlife is poorly defined in Cameroon. As wildlife could be a reservoir host of bTB; susceptibility of Cameroonian antelope and other wildlife species, such as buffalo and warthogs, to *M*. *bovis* requires further investigation [58].

These observations on cattle husbandry assist in identifying priorities for bTB research in this environment. Biologically, there is a need to understand the relative importance of the different routes of transmission and the susceptibility and infection status of potential reservoir hosts. Socio-economically there is a need to understand the drivers for transhumance as there appeared to be geographical and ethnic differences in the practice between communities. Highlighting a potential problem if disease free zones were to be established where transhumance is undertaken. The reasons for this difference in grazing practices is unclear. It may reflect pressure on land or different cultural traditions. Conflict between pastoralists and arable farmers in the NWR has been recognised as a perennial problem and the subject of a number of studies of the competition for the natural resources of land and water [59–62]. Cultural difference in the nomadic activities of the Mbororo and sedentary Fulbe are also well recognised [29]. But there is evidence that the frequency of transhumance is declining. In a previous study of pastoralists in the VD carried out in 2000, 29.2% reported transhumance compared with 6% in the current study which would be of benefit for bTB control [63].

In addition to understanding cattle demography and contact networks, the awareness and knowledge of pastoralists and small-scale dairy farmers about bTB will be an important component of any bTB control scheme. Awareness of bTB amongst pastoralists was associated with Region with a greater portion being aware in the NWR than VD. This regional difference was surprising. It may be because pastoralists had worked with cattle for longer in the NWR than the VD. However this seems unlikely because all pastoralists were aware of FMD and fasciolosis, endemic diseases in both sites, and there was no difference in awareness between groups. It is possible that they encountered bTB more frequently in the NWR either from clinical cases or abattoir condemnations. A previous abattoir study reports a higher bTB prevalence in the NWR than the VD potentially supporting this theory[25]. Also a similar proportion of pastoralists, who were aware of bTB, in each area were able to identify clinical signs consistent with bTB; such as coughing, weight loss, poor coat and weakness. It is important to note that, unlike FMD where the clinical signs are relatively pathognomonic, that clinical signs of bTB may not develop in an infected animal despite the presence of severe pathology [2,64,65]. Yet detections of TB lesions in abattoirs may raise awareness as pastoralists are notified of carcase condemnations at abattoirs, as the vending herdsman and purchasing butcher share the financial loss of the condemnation. Furthermore veterinary staff, inspecting slaughtered cattle, may inform herdsmen about clinical signs of bTB and subsequently increase awareness.

Awareness of bTB amongst dairy farmers was associated with gender, with male dairy farmers 6 times more likely to be aware of bTB than females. Interestingly this pattern was repeated for the other diseases, with male dairy farmers also more aware of fasciolosis and FMD. Potentially women did not benefit from the cattle husbandry training given when dairy cooperatives and the reasons for this are unclear. In other low income countries, were there is poor knowledge of livestock diseases, it has been shown that education programs have not targeted the primary individuals involved in livestock rearing [66]. Other studies have shown that livestock education programs are often directed at men as they are presumed to be the individual primarily involved in livestock production [67]. Yet this is not the case across all livestock rearing communities and this is demonstrated in Cameroon with half of dairy farmers being female. Taking into account the strong movement to encourage women into livestock keeping, as a method of poverty alleviation, it is important that any future bTB educational programs are not gender biased [68]. Interestingly dairy farmers appeared to be less able to identify clinical signs of bTB than pastoralists. This was also a consistent trend for fasciolosis and to a lesser extent FMD. It is unclear why such a high percentage of farmers who had heard of these diseases were unaware of their clinical signs. It may reflect the frequency of exposure of dairy farmers to these diseases with dairy cattle being managed as individuals with little mixing with other cattle. Reasons for inconsistencies in bTB awareness are unclear and further research is required in this area and may hinder the acceptance of future control programs in certain communities.

One of the main reasons for controlling bTB is due to its potential of being a milk-borne zoonosis [6,13]. Unsurprisingly, with regular access to milk, there appears to be widespread milk consumption and processing of milk in pastoralist and dairy farmer’s families. About half of the dairy farmers were aware of milk borne disease but interestingly few were aware of milk borne transmission of *M*. *bovis*. The proportion of pastoralists who were aware of zoonotic TB transmission in milk was even lower than dairy farmers. This suggests that improving knowledge and awareness of milk borne transmission is an important message in any public health program to control zoonotic TB. Transmission of zoonotic TB, through consumption of infected milk, can be controlled by heating milk for example through pasteurisation [69]. Although the prevalence of heating milk was collected in this study we have no information on the temperature to which it was heated e.g. it is possible that it was always heated to a sufficient temperature and time to destroy *M*. *bovis*. Data on the duration of heating were collected but in the absence of information on the volume and temperature of milk being heated these are difficult to interpret. However there were some interesting differences in the patterns of heating. All dairy farmers heated milk for their families but just over a quarter heated milk prior to sale. As most dairy farmers were producing milk for non-family members this represents an important potential route of transmission of bTB. It is unclear why this difference should exist in milk consumed by the family and by non-family members. It may reflect the financial cost of purchasing fuel to heat the milk or a preference by consumers for raw milk e.g. because of ease in detecting its freshness or because they wish to preserve milk by converting it into yogurt or cheese. Pastoralists also heated milk for family consumption. In contrast to dairy farmers, fewer pastoralists produced milk for non-family members especially in the VD. This difference may reflect the recognition by pastoralists in the NWR of the financial value of the developing dairy market in the Region. A similar percentage of NWR pastoralist and dairy farmers used artificial insemination, with Holstein-Friesian semen, compared to VD pastoralists. Suggesting an overall drive for increased milk production by both cattle rearing groups in the NWR. In addition to heating, souring milk was common amongst pastoralists, especially in the VD, appearing to be not culturally important to dairy farmers preserving milk. There are limited and mixed reports of the efficacy of traditional milk souring techniques to destroy *M*. *bovis* [70–72]. A study in Zambia showed milk spiked with *M*. *bovis* and then naturally soured over 1–3 days did not consistently destroy the bacterium [73]. However, a recent study from South Africa suggests that souring is effective if the product is maintained at an adequate temperature and is time dependent [74]. Additionally efficacy of souring may dependent upon pH reached and the bacterial populations [75,76]. Hence the final soured product varies with different souring techniques and souring methods vary across sub-Saharan Africa, In this study pastoralists soured milk for a median of one day. Souring for one to two days, without a starter culture, has also been previously reported in Cameroon but it is unclear if the same techniques are used homogenously across the country [32,75]. Unlike heating, souring requires no external energy source or additional financial investment so maybe useful in low-income settings if done correctly [74]. Hence identifying the variety of souring techniques, their efficacy and awareness could inform future public health education programs.

In conclusion, this study has described the cattle husbandry, dairy practices and knowledge of bTB in pastoralist and small-scale dairy farming communities. The presence of different cattle rearing systems within a country, pose different challenges to be taken into account when developing bTB control programs. The study has also identified a need for investigation of current milk processing practices to determine whether they are effective in inactivating *M*. *bovis* in countries where milk processing is unregulated. Overall the gap in bTB knowledge and awareness identified may hinder future *M*. *bovis* control in cattle and people. Looking to the future targeted TB education programs within cattle rearing communities could be potentially beneficial to raise the awareness of zTB and improve peoples understanding of mitigating actions such as boiling and souring milk.

## Supporting Information

S1 File FieldQuestionnaire_PLOSone.pdf. Questionnaire, in Fulfulde language, used in the pastoral and dairy cross-sectional studies.
(PDF)

S2 File DescriptiveStatistics_PLOSone.docx. Reference table of descriptive data analysis from pastoral and dairy cross-sectional studies.
(DOCX)
