# Supplementary material for: Knowledge of Bovine Tuberculosis, Cattle Husbandry and Dairy Practices amongst Pastoralists and Small-Scale Dairy Farmers in Cameroon
Source: PLoS One. 2016 Jan 8;11(1):e0146538. doi: 10.1371/journal.pone.0146538 (PMC4706344; doi:10.1371/journal.pone.0146538)
Supplement: S1 File — Questionnaire, in Fulfulde language, used in the pastoral and dairy cross-sectional studies. (PDF) [file pone.0146538.s001.pdf]

CAMbTB Cameroon Cattle Herder Field Study Questionnaire

Introduction to the Project:

Instructions for administering this questionnaire

1) Ask the questions as written. Remember the questionnaire is a measurement tool in the same way as a set of scales and consistency is critical. If prompting is necessary keep to a minimum and always use the same statement for each question. **Please ask all questions highlighted in grey unless not applicable; then refer to highlighted instructions to direct you to the next question to ask.**
2) Do not rush the interviewee, do not give any indication of your opinion, i.e. maintain a neutral expression, but show your interest.
3) Find somewhere private to conduct the interview away from any officials or neighbours who might affect the herdsman responses.
4) Remember **ALL ANSWERS ARE CONFIDENTIAL AS IS THE NAME OF THE INTERVIEWEE** and if another herdsman asks if his friend is included or about any of his responses you politely reply that you are unable to answer because we have promised everyone that the information is confidential. You need not say anything more.
5) In the event that a herdsman does not want to participate it is important at least to get the background details to allow us to classify the non-responders.
6) Before starting the questionnaire read the following statement.

"Thank you for agreeing to participate in this study of bovine tuberculosis, Liver Fluke/fasciolosis and other diseases in Cameroon. The project's purpose is to gain further understanding about various infectious diseases in Cameroonian cattle. The information you give will be used to understand why infections spread and help improve control of these diseases. Hopefully benefiting livelihoods by improving cattle health and production.

You have been selected by a random process from a list of names of people who had their herd vaccinated in the last 2 years. The choice of your herd in no way suggests there is anything wrong with your cows or other reasons. It is a choice just like the game of lottery. The names were put on the computer that then chose the names.

We now want to ask you some questions about how you manage **this particular herd you have here today**. This will help us assess various aspects of disease transmission and help in the interpretation of the results from the blood samples, and so give better advice to all herdsmen. All answers will be kept confidential and your answers will not be given to any other group. Your name will not be used in any report and only summary statistics will be quoted.

If you happy with this we would like to begin the questionnaire."

If verbal consent is granted then continue with the questionnaire.

Date: ___________________

Herd ID: __ __ __ / __ __ __

General Herd Information:

(0.0) GPS (*Circle*)	**Y**	**N**	**UNK**

(0.01) N			.						(0.02) E				.					(0.03) ALT	

(0.04) ZVSCC		(0.05) ZVSCC Code	

(0.06) Village		(0.07) Div		(0.08) SubDiv	

(0.09) Number of cattle presented		(0.10) Number of cattle sampled		(0.11) Interviewer	

(0.12) Additional comments	
If questionnaire stopped prior completion: reason why and question stopped at.	
(0.13) Question stopped at:	

1. Herdsman Information:

1.01 What is your full name?	

1.02 Who are you in regard to the cattle presented? (*Circle one*)				
**Owner**	**Herdsman**	**Caretaker**	**Other:** ____________	**UNK**

1.03 How many years have you worked with cattle?	____________ **Years**	**UNK**

1.04 What is your ethnic group?	

1.05 What is your education level? (*Circle one*)	**NONE**	**PRIMARY**	**SECONDARY**	**HIGHER**	**UNK**

1.06 How old are you?	____________ **Years**	**UNK**

1.07 Record the owner's gender. (*Circle one*)	**M**	**F**

1.08 How many years have you kept cattle in this area?	____________ **Years**	**UNK**

What other animals, other than cattle, do you currently keep/ rear at your homestead? (*Circle one per row*)			
(1.09) Sheep	**Y**	**N**	**UNK**
(1.10) Goats	**Y**	**N**	**UNK**
(1.11) Poultry	**Y**	**N**	**UNK**
(1.12) Cats	**Y**	**N**	**UNK**
(1.13) Dogs	**Y**	**N**	**UNK**
(1.14) Horses	**Y**	**N**	**UNK**
(1.15) Other	____________	**N**	**UNK**

2. Infectious Diseases:

This first section is about your perception of diseases you may have encountered and possibly have in your herd.

--------------------------------FOOT AND MOUTH DISEASE----------------------------

2.01 Are you aware of a disease called "Foot and Mouth Disease"? (*Circle one*)	**Y**	**N**	**UNK**
If no or unknown, go to question 2.06			

2.02 What clinical signs do you associate with foot and mouth disease in cattle? *(Do not read the options and tick all as appropriate)*			
Weakness		Weight Loss	
Inappetence		Diarrhoea	
Coughing all the time		Breathing Difficulties	
Coughing intermittently		Bottle Jaw/ Neck Swelling	
Nasal Discharge		Lameness	
Recumbent		Enlarged Lymph Nodes	
Infertility		Aggression	
Mastitis/ Enlarged Udder		Reduced Milk Yield	
Eye Problems		Salivation/ Drooling	
Death		Arched Back	
Blood in Urine		Abortion	
Poor Coat		Separates from group	
Will not Breed		Swollen Testicles	
Other:		Does not know any clinical signs	

2.03 Have any of the cattle presented been sick from foot and mouth disease in the past 12 months? (*Circle one*)		
**Y**	**N**	**UNK**

2.04 Have any of your cattle died from foot and mouth disease in the past 12 months? (*Circle one*)		
**Y**	**N**	**UNK**
If no or unknown, go to question 2.06		

2.05 **If yes**, how many animals have died from foot and mouth disease in the past 12 months? (*Circle one*)					
**0 cattle**	**1-5 cattle**	**6-10 cattle**	**11-15 cattle**	**15+ cattle**	**UNK**

------------------------------------BOVINE TUBERCULOSIS---------------------------------

2.06 Are you aware of a disease called "Bovine Tuberculosis"? (*Circle one*)	**Y**	**N**	**UNK**
If no or unknown, go to question 2.18			

2.07 What clinical signs do you associate with bovine tuberculosis in cattle? *(Do not read the options and tick all as appropriate)*			
Weakness		Weight Loss	
Inappetence		Diarrhoea	
Coughing all the time		Breathing Difficulties	
Coughing intermittently		Bottle Jaw/ Neck Swelling	
Nasal Discharge		Lameness	
Recumbent		Enlarged Lymph Nodes	
Infertility		Aggression	
Mastitis/ Enlarged Udder		Reduced Milk Yield	
Eye Problems		Salivation/ Drooling	
Death		Arched Back	
Blood in Urine		Abortion	
Poor Coat		Separates from group	
Will not Breed		Swollen Testicles	
Other:		Does not know any clinical signs	

2.08 Have any of the cattle presented been sick from bovine tuberculosis, not including cattle that have died, in the past 12 months? (*Circle one*)		
**Y**	**N**	**UNK**

2.09 Have any of your cattle died from bovine tuberculosis in the past 12 months? (*Circle*)		
**Y**	**N**	**UNK**

2.10 How many animals have died from bovine tuberculosis in the past 12 months? (*Circle one*)					
**0 cattle**	**1-5 cattle**	**6-10 cattle**	**11-15 cattle**	**15+ cattle**	**UNK**

2.11 Have you been informed of any your cattle sold or slaughtered have bovine tuberculosis in the past 12 months? (*Circle one*)		
**Y**	**N**	**UNK**

2.12 Have you EVER been informed of any your cattle sold or slaughtered have bovine tuberculosis? (*Circle one*)		
**Y**	**N**	**UNK**

2.13 Have any of your cattle been tested for bovine tuberculosis? (*Circle one*)		
**Y**	**N**	**UNK**
If no or unknown, go to question 2.18		

2.14 **If yes,** how many months ago were they last tested for bovine tuberculosis?	____________________**Months**

2.15 Were any bovine tuberculosis positive cattle reported on this test? (*Circle one*)		
**Y**	**N**	**UNK**
If no or unknown, go to question 2.18		

2.16 **If yes,** how many cattle tested positive to bovine tuberculosis? (*Circle one*)					
**0 cattle**	**1-5 cattle**	**6-10 cattle**	**11-15 cattle**	**15+ cattle**	**UNK**

2,17 What was done with test positive cattle? (*Free text*)	

--FASCIOLOSIS---------------------------------------

2.18 Are you aware of a disease called "Liver Fluke or Fasciolosis"? (*Circle one*)	**Y**	**N**	**UNK**
If no or unknown, go to question 2.25			

2.19 What clinical signs do you associate with Liver Fluke or Fasciolosis in cattle? *(Do not read the options and tick all as appropriate)*			
Weakness		Weight Loss	
Inappetence		Diarrhoea	
Coughing all the time		Breathing Difficulties	
Coughing intermittently		Bottle Jaw/ Neck Swelling	
Nasal Discharge		Lameness	
Recumbent		Enlarged Lymph Nodes	
Infertility		Aggression	
Mastitis/ Enlarged Udder		Reduced Milk Yield	
Eye Problems		Salivation/ Drooling	
Death		Arched Back	
Blood in Urine		Abortion	
Poor Coat		Separates from group	
Will not Breed		Swollen Testicles	
Other:		Does not know any clinical signs	

2.20 Have any of the cattle presented been sick from liver fluke infection in the past 12 months? (*Circle one*)		
**Y**	**N**	**UNK**

2.21 Have any of your cattle died from liver fluke infection in the past 12 months? (*Circle one*)		
**Y**	**N**	**UNK**

2.22 **If yes**, how many animals have died from liver fluke infection in the past 12 months? (*Circle one*)					
**0 cattle**	**1-5 cattle**	**6-10 cattle**	**11-15 cattle**	**15+ cattle**	**UNK**

2.23 Have you been informed of any your cattle sold or slaughtered have liver fluke infection in the past 12 months? (*Circle one*)		
**Y**	**N**	**UNK**

2.24 Have you EVER been informed of any your cattle sold or slaughtered have liver fluke infection? (*Circle one*)		
**Y**	**N**	**UNK**

2.25 Are you aware of any other major health concerns that have affected your cattle in the past 12 months? (*Free text*)

3. Routine Health Care:

The next set of questions asks you about if you provide veterinary care for your herd.

3.01 Have the cattle presented been vaccinated in the previous 12 months? (*Circle one*)		
**Y**	**N**	**UNK**

3.02 Have you treated the cattle presented with an anthelmintic/ wormer in the previous 12 months? (*Circle one*)		
**Y**	**N**	**UNK**
If no or unknown, go to question 3.06		

3.03 **If yes,** what was the name of the drug used? (*Free text*)

3.04 **If yes,** which cattle do you select to be treated? (*Circle multiple appropriate answers*)			
**ALL**	**SICK**	**NONE**	**UNK**

3.05 **If yes,** which age groups of cattle were treated? *(Circle multiple appropriate answers)*			
**Calves (0-1 years)**	**Young Stock (1-3 years)**	**Adult (3+ years)**	**UNK**

3.06 Have you treated the cattle presented for trypanosomiasis in the previous 12 months? (*Circle one*)		
**Y**	**N**	**UNK**
If no or unknown, go to question 4.01		

3.07 **If yes,** what was the name of the drug used? (*Free text*)

3.08 **If yes,** which cattle did you select to be treated? (*Circle one*)			
**ALL**	**SICK**	**NONE**	**UNK**

3.09 If yes, which age groups of cattle were treated? *(Circle multiple appropriate answers)*			
**Calves (0-1 years)**	**Young Stock (1-3 years)**	**Adult (3+ years)**	**UNK**

4. Reproduction

The next questions are about how you manage cattle breeding in your herd.

4.01 Have you used natural breeding in the previous 12 months? (*Circle one*)		
**Y**	**N**	**UNK**
If no or unknown, go to question 4.03		

4.02 What breed of bull is used for natural breeding? (*Free text*)

4.03 Have you used Artificial Insemination (AI) in the previous 12 months? (*Circle one*)		
**Y**	**N**	**UNK**
If no or unknown, go to question 5.01		

4.04 What breed of bull is used for Artificial Insemination (AI)? (*Free text*)

5. Grazing and Housing:

The next sets of questions are about how you manage grazing and the nutrition of these animals presented. This section does NOT regard transhumance as this will be discussed in a later section.

5.01 Have the cattle presented been allowed to graze in open pasture the in previous 12 months? (*Circle one*)		
**Y**	**N**	**UNK**
If no or unknown, go to question 5.09		

5.02 In the area regularly grazed by these cattle presented, is it (*Circle one*):		
**Natural**	**Improved**	**UNK**
If unknown, go to question 5.09		

5.03 Are any parts of the pasture grazed by these cattle presented, in the past 12 months, flooded or swampy? (*Circle one*)		
**Y**	**N**	**UNK**

5.04 How many other herds graze the same pasture, as these cattle, on a daily basis? (*Circle one*)					
**0 herds**	**1-5 herds**	**6-10 herds**	**11-15 herds**	**15+ herds**	**UNK**

Do any of these cattle presented come in contact with the following wild animals whilst grazing? (*Circle one per row*)			
(5.05) Buffalo	**Y**	**N**	**UNK**
(5.06) Antelope or deer	**Y**	**N**	**UNK**
(5.07) Warthog	**Y**	**N**	**UNK**
(6.08) Other	______________	**N**	**UNK**

5.09 Have the presented cattle been kept housed the majority of their time in the past 12 months?(*Circle one*)		
**Y**	**N**	**UNK**

5.10 Do you keep these cattle, presented, in a fenced enclosure over night? (*Circle one*)		
**Y**	**N**	**UNK**
If no or unknown, go to question 5.13		

5.11 **If yes,** do the cattle presented share the fenced enclosure with other herds? (*Circle one*)		
**Y**	**N**	**UNK**

5.12 How many other herds do your cattle share the fenced enclosure with? (*Circle one*)					
**0 herds**	**1-5 herds**	**6-10 herds**	**11-15 herds**	**15+ herds**	**UNK**

5.13 Have you fed these cattle presented any other supplements in the previous 12 months? (*Circle one*)		
**Y**	**N**	**UNK**
If no or unknown, go to question 5.15		

5.14 **If yes,** what did you feed? (*Free text*)

Which of the following do the presented cattle drink from on a regular basis ?: (*Circle one per row*)			
(5.15) Water troughs	**Y**	**N**	**UNK**
(5.16) Water canals	**Y**	**N**	**UNK**
(5.17) Streams	**Y**	**N**	**UNK**
(5.18) Lakes or Ponds	**Y**	**N**	**UNK**

5.19 How many other herds do your cattle contact at these watering points regulary? (*Circle one*)					
**0 herds**	**1-5 herds**	**6-10 herds**	**11-15 herds**	**15+ herds**	**UNK**

5.20 How many other herds use the same watering points as your cattle? (*Circle one*)					
**0 herds**	**1-5 herds**	**6-10 herds**	**11-15 herds**	**15+ herds**	**UNK**

6. Transhumance:

The next series of questions relate only when your herd go on transhumance, if this is applicable.

6.01 Did any of the cattle presented go on transhumance in the past 12 months? (*Circle one*)		
**Y**	**N**	**UNK**
If no or unknown, go to question 7.01		

6.02 What is the name of the area your cattle go to on transhumance?					
**Area**		**Div**		**SubDiv**	
**UNK**					

6.03 How many days walk from where the cattle are now is is?	
____________________**Days**	**UNK**

6.04 Have the cattle presented; grazed on flooded or swampy pasture whilst on transhumance in the past 12 months? (*Circle one*)		
**Y**	**N**	**UNK**

6.05 How many herds on average does your herd contact on a daily basis in the transhumance area? (*Circle one*)					
**0 herds**	**1-5 herds**	**6-10 herds**	**11-15 herds**	**15+ herds**	**UNK**

Do any of these cattle presented come in contact with the following wild animals whilst on transhumance? (*Circle one per row*)			
(6.06) Buffalo	**Y**	**N**	**UNK**
(6.07) Antelope or deer	**Y**	**N**	**UNK**
(6.08) Warthog	**Y**	**N**	**UNK**
(6.09) Other	____________	**N**	**UNK**

6.10 Which month did you go on transhumance? (*Circle as appropriate*)												
**Jan**	**Feb**	**Mar**	**Apr**	**May**	**Jun**	**Jul**	**Aug**	**Sep**	**Oct**	**Nov**	**Dec**	**UNK**

6.11 Which month did you return from transhumance? (*Circle as appropriate*)												
**Jan**	**Feb**	**Mar**	**Apr**	**May**	**Jun**	**Jul**	**Aug**	**Sep**	**Oct**	**Nov**	**Dec**	**UNK**

7. Cattle Sales:

The next series of questions are about purchase and sale of cattle.

7.01 Have you sold any cattle in the past 12 months? (*Circle one*)		
**Y**	**N**	**UNK**
If no or unknown, go to question 7.03		

7.02 How many times have you sold cattle in the past 12 months? (*Circle one*)

7.03 Have you bought any cattle in the past 12 months? (*Circle one*)		
**Y**	**N**	**UNK**
If no or unknown, go to question 7.05. If no or unknown, to both 7.01 and 7.03, go to question 8.01		

7.04 How many times have you purchased cattle in the past 12 months? (*Free text*)

7.05 Have sales or purchases been conducted at these places or with the following people in the past 12 months (*Circle multiple appropriate answers*)						
**Markets**	**"Buyem and sellems"**	**Breeder**	**Neighbour**	**Other Herder**	**OTHER** ___________	**UNK**

8. Milk and Dairy Habits:

The final set of questions are about milking your cows and drinking their milk.

8.01 Do you or any of your family drink milk from these animals presented? (*Circle one*)		
**Y**	**N**	**UNK**
If no or unknown, go to question 8.07		

8.02 Do you treat or heat this milk drunk by your family? (*Circle one*)		
**Y**	**N**	**UNK**
If no or unknown, go to question 8.07		

8.03 Do you treat this milk by souring? (*Circle one*)		
**Y**	**N**	**UNK**
If no or unknown, go to question 8.05		

8.04 **If yes,** how long is milk soured for?	______________**days**	**UNK**
If no or unknown, go to question 8.05		

8.05 Do you treat this milk by heating? (*Circle one*)		
**Y**	**N**	**UNK**
If no or unknown, go to question 8.07		

8.06 **If yes,** how long is milk heated for?	___________**minutes**	**UNK**

8.07 Is the milk from these animals presented sold or given to other people other than your family? (*Circle one*)		
**Y**	**N**	**UNK**
If no or unknown, go to question 8.13		

8.08 Do you treat or heat the milk sold or given to other people? (*Circle one*)		
**Y**	**N**	**UNK**
If no or unknown, go to question 8.13		

9.09 Do you treat this milk by souring? (*Circle one*)		
**Y**	**N**	**UNK**
If no or unknown, go to question 8.11		

8.10 **If yes,** how long is this milk soured for?	______________**days**	**UNK**
If no or unknown, go to question 8.11		

8.11 Do you treat this milk by heating? (*Circle one*)		
**Y**	**N**	**UNK**
If no or unknown, go to question 8.13		

8.12 **If yes,** how long is this milk heated for?	____________**Minutes**	**UNK**

8.13 Is any of your milk mixed with milk from other herds prior treatment or heating? (*Circle one*)			
**Y**	**N**	**UNK**	**NA**

8.14 Do you produce any of the following dairy products from your cow's milk? (*Circle multiple appropriate answers*)					
**Cheese**	**Butter**	**Yoghurt**	**Other:** ____________	**N**	**UNK**
If no or unknown, go to question 8.17					

8.15 **If yes,** do you or any of your family consume any of these dairy products? (*Circle one*)		
**Y**	**N**	**UNK**

8.16 **If yes,** do you sell or give any of these dairy products to other people other than your family?		
**Y**	**N**	**UNK**

8.17 Do you know of any diseases which people can get from drinking cow's milk? (*Circle one*)		
**Y**	**N**	**UNK**
If no or unknown, go to question 8.19		

8.18 What are the names of these diseases? (*Free text*)

8.19 Do you have any additional comments you wish to make about the questionnaire or the subjects covered?(*Free text, continue on other side if needed*)

Thank you very much for taking time to answer our questions, we will be in touch with our findings.
