# Supplementary material for: Knowledge of Bovine Tuberculosis, Cattle Husbandry and Dairy Practices amongst Pastoralists and Small-Scale Dairy Farmers in Cameroon
Source: PLoS One. 2016 Jan 8;11(1):e0146538. doi: 10.1371/journal.pone.0146538 (PMC4706344; doi:10.1371/journal.pone.0146538)
Supplement: S2 File — Reference table of descriptive data analysis from pastoral and dairy cross-sectional studies. (DOCX) [file pone.0146538.s002.docx]

**Reference tables of descriptive statistics from manuscript:**

*"****Knowledge of bovine tuberculosis, cattle husbandry and dairy practices amongst pastoralists and small-scale dairy farmers in Cameroon****."*

*R Kelly et al. 2015*

**Table 1. A descriptive summary of cattle keeper information in pastoral and dairy cattle from cross-sectional studies.**

|  | North West Region  Pastoralists  (95% CI, n=50) | | | | Vina Division  Pastoralists  (95% CI, n=50) | | | North West Region  Dairy Farmers  (95% CI, n=46) | |
| --- | --- | --- | --- | --- | --- | --- | --- | --- | --- |
| PARTICIPANTS | | | | | | | | | |
| Gender | | | | | | | | | |
| *Male* | **97.8%** (86.4-99.7%) | | | | **100%** (92.9-100%) | | | **56.5%** (42.0-71.0%) | |
| *Female* | **2.2%** (0.0-11.6%) | | | | **0.0%** (0.0-7.1%) | | | **43.5%** (29.0-58.0%) | |
| Age | | | | | | | | | |
| Mean (Years) | **41.0** (37.0-44.9) | | | | **39.2** (35.3-43.4) | | | **45.8** (42.4-49.3) | |
| Schooling level | | | | | | | | | |
| *None* | **63.2%** (50.0-74.7%) | | | | **74.0%** (60.6-84.2%) | | | **0%** (0.0-7.7%) | |
| *Primary* | **24.8%** (14.9-38.4%) | | | | **23.9%** (14.1-37.4%) | | | **76.1%** (63.3-88.5%) | |
| *Secondary* | **8.1%** (3.1-19.2%) | | | | **2.0%** (0.2-12.3%) | | | **10.9%** (1.8-20.0%) | |
| *Higher* | **3.9%** (0.1-13.0%) | | | | **0.0%** (0.0-7.1%)) | | | **5.5%** (4.0-6.9%) | |
| Mean time kept cattle (Years) | **26.5** (22.4-30.5) | | | | **17.7** (13.7-21.4) | | | **5.5** (4.0-6.9) | |
| Ethnic group | | | | | | | | | |
| *Mbororo* | **89.4%** (77.4-95.4%) | | | | **17.6%** (9.9-29.5%) | | | **0.0%** (0.0-7.7%)) | |
| *Fulbe* | **2.0%** (0.2-12.7%) | | | | **66.1%** (53.0-77.1%) | | | **0.0%** (0.0-7.7%)) | |
| *Other** | **8.6%** (3.3-20.2%) | | | | **16.2%** (8.3-29.2%) | | | **100%** (92.3-100%) | |
| Cattle keeper type | | | | | | | | | |
| *Caretaker* | **20.2%** (11.9-32.4%) | | | | **27.5%** (17.2-40.9%) | | | **6.5%** (0.0-13.7%) | |
| *Herdsman* | **31.9%** (20.3-46.2%) | | | | **45.5%** (34.5-60.8%) | | | **0.0%** (0.0-7.7%) | |
| *Owner* | **47.9%** (35.3-60.8%) | | | | **27.5%** (17.2-40.9%) | | | **93.5%** (86.3-100%) | |
| Length of time kept cattle | | | | | | | | | |
| *Mean (Years)* | **26.5** (22.4-30.5) | | | | **17.7** (13.7-21.4) | | | **5.5** (4.0-6.9) | |
| HERD | | | | | | | | | |
| Size | | | | | | | | | |
| *Mean* | **50** (45-55) | | | | **38** (34-43) | | | **3** (2-3) | |
| Breeds kept | | | | | | | | | |
| *Mixed* | **63.9%** (54.6-72.2%) | | | | **15.0%** (9.7-30.1%) | | | **0.0%** (0.0-7.7%) | |
| *Red Fulani* | **16.1%** (10.1-24.7%) | | | | **0.0%** (0.0-7.1%) | | | **0.0%** (0.0-7.7%) | |
| *White Fulani* | **20.0%**  (12.9-29.8%) | | | | **1.5%** (0.2-1.0%) | | | **0.0%** (0.0-7.7%) | |
| *Gudali* | **0.0%** (0.0-7.1%) | | | | **83.5%** (78.1-88.8%) | | | **0.0%** (0.0-7.7%) | |
| *Holstein Friesian* | **0.0%** (0.0-7.1%) | | | | **0.0%** (0.0-7.1%) | | | **100%** (92.3-100%) | |
| OTHER SPECIES | | | | | | | | | |
| What other animals, other than cattle, do you currently keep or rear at your homested? | | | | | | | | | |
| *Sheep* | | **45.2%** (33.7-57.1%) | | **28.8%** (18.7-41.6%) | | | **23.9%** (11.5-36.4%) | | |
| *Goats* | | **29.9%** (19.2-43.4%) | | **27.2% (**16.7-41.0%) | | | **30.4%** (20.9-48.7%) | | |
| *Poultry* | | **75.4%** (61.2-85.7%) | | **65.4%** (51.1-77.4%) | | | **63.0%** (48.9-77.1%) | | |
| *Cats* | | **42.9%** (30.7-55.9%) | | **43.4%** (31.2-56.6%) | | | **26.0%** (13.3-38.9%) | | |
| *Dogs* | | **36.4%** (24.5-50.5%) | | **31.3%** (20.5-44.5%) | | | **54.3%** (42.0-71.0%) | | |
| *Horses^* | | **39.9%** (28.2-53.0%) | | **2.0%** (0.2-12.4%) | | | **2.2%** (0.0-6.4%) | | |
| Do any of these cattle presented come in contact with the following wild animals whilst grazing? | | | | | | | | | |
| *Buffalo* | | **0.0%** (0.0-7.1%) | | **0.0%** (0.0-7.1%) | | | **NA** | | |
| *Antelope* | | **49.8%** (36.0-63.6%) | | **76.4%** (65.4-84.7%) | | | **NA** | | |
| *Warthog* | | **11.9%** (5.4-24.0%) | | **38.2%** (27.2-50.6%) | | | **NA** | | |
| If you cattle go on transhumance; do any of these cattle presented come in contact with the following wild animals whilst on transhumance? | | | | | | | | | |
|  | | (n=22) | | (n=3) | | | NA | | |
| *Buffalo* | | **27.3%** (12.9-48.8%) | | **0.0%** (0.0-70.8%) | | | **NA** | | |
| *Antelope* | | **81.8%** (60.6-92.9%) | | **66.7%** (16.3-95.3%) | | | **NA** | | |
| *Warthog* | | **31.8%** (16.1-53.2%) | | **66.7%** (16.3-95.3%) | | | **NA** | | |
| CATTLE INFECTIOUS DISEASE CONTROL | | | | | | | | | |
| Have the cattle presented been vaccinated? | | | | | | | | | |
| *Yes* | | | **98.0%** (87.6-99.7%) | | | **100%** (92.9-100%) | | | **97.8%** (93.6-100%) |
| Have you treated the cattle presented with an anthelmintic? | | | | | | | | | |
| *Yes* | | | **93.9%** (82.6-98.1%) | | | **84.2%** (71.2-92.0%) | | | **100%** (92.3-100%) |
| Have you treated any of the cattle presented for trypanosomiasis? | | | | | | | | | |
| *Yes* | | | **41.9%** (29.1-56.0%) | | | **77.7%** (65.9-86.2%) | | | **0.0%** (0.0-8.6%) |
| CATTLE REPRODUCTION PRACTICES | | | | | | | | | |
| Have you used natural breeding? | | | | | | | | | |
| *Yes* | | | **100%** (92.9-100%) | | | **100%** (92.9-100%) | | | **89.1%** (80.0-98.2%) |
| If so; What breed of bull do you use for natural breeding? | | | (n=50) | | | (n=50) | | | (n=41) |
| *Fulani* | | | **39.6%** (26.7-52.5%) | | | **0.0%** (0.0-7.1%) | | | **0.0%** (0.0-8.6%) |
| *Gudali* | | | **0.0%** (0.0-7.1%) | | | **91.4%** (85.5-97.2%) | | | **0.0%** (0.0-8.6%) |
| *Mixed Breed* | | | **62.5%** (50.3-74.7%) | | | **5.4%** (0.0-11.3%) | | | **0.0%** (0.0-8.6%) |
| *Holstein-Friesian* | | | **0.0%** (0.0-7.1%) | | | **0.0%** (0.0-7.1%) | | | **100%** (91.4-100%) |
| Have you used artificial insemination (AI)? | | |  | | |  | | |  |
| *Yes* | | | **10.2%** (4.4-22.01%) | | | **2.0%** (0.0-5.8%) | | | **8.0%** (1.7-14.4%) |
| If so; What breed of bull do you use for AI? | | | (n=5) | | | (n=1) | | | (n=5) |
| *European Breed*  *(Bos taurus)* | | | **100%** (47.8-100%) | | | **100%** (2.5-100%) | | | **100%** (47.8-100%) |
| CATTLE GRAZING AND HOUSING PRACTICES | | | | | | | | | |
| Have the presented cattle been kept housed the majority of time? | | | | | | | | | |
| *Yes* | | | **0.0%** (0.0-7.1%) | | | **0.0%** (0.0-7.1%) | | | **97.8%** (93.6-100%) |
| Have these cattle grazed open pasture? | | | | | | | | | |
| *Yes* | | | **100%** (92.9-100%) | | | **100%** (92.9-100%) | | | **4.35%** (0.0-10.3%) |
| Do you keep these cattle presented in a fenced enclosure overnight? | | | | | | | | | |
| *Yes* | | | **54.7%** (43.1-66.3%) | | | **17.1%** (8.8-25.3%) | | | **100%** (15.8-100%) |
| If so; What type of pasture do these cattle graze? | | | (n=50) | | | (n=50) | | | (n=2) |
| *Natural pasture* | | | **98.7%** (96.1-100%) | | | **100%** (92.9-100%) | | | **0.0%** (0.0-84.2%) |
| *Improved pasture* | | | **1.3%** (0.0-3.9%) | | | **0.0%** (0.0-7.1%) | | | **100%** (15.8-100%) |
| Where do the presented cattle drink from on a regular basis? | | | | | | | | | |
| *Streams* | | | **97.9%** (86.6-99.7%) | | | **97.9%** (86.6-99.7%) | | | **0.0%** (0.0-8.6%) |
| *Water troughs* | | | **0.0%** (0.0-7.1%) | | | **0.0%** (0.0-7.1%) | | | **100%** (92.3-100%) |
| *Water canals* | | | **1.9%** (0.3-12.3%) | | | **0.0%** (0.0-7.1%) | | | **0.0%** (0.0-8.6%) |
| *Lakes and ponds* | | | **1.9%** (0.3-12.3%) | | | **0.0%** (0.0-7.1%) | | | **0.0%** (0.0-8.6%) |
| Did any of these cattle presented go on transhumance? | | | | | | | | | |
| *Yes* | | | **43.8%** (31.4-57.1%) | | | **6.2%** (2.0-17.7%) | | | **0.0%** (0.0-8.6%) |
| CATTLE TRADE PRACTICES | | | | | | | | | |
| Did you in the past 12 months: | | | | | | | | | |
| *Purchase cattle?* | | | **41.8%** (30.0-54.7%) | | | **49.7%** (36.4-62.9%) | | | **8.7%** (0.4-16.9%) |
| *Sell cattle?* | | | **93.8%** (83.2-97.9%) | | | **83.9%** (71.3-91.6%) | | | **37.0%** (22.9-51.1%) |
| If purchased or sold cattle did you do so at markets? | | | (n=42) | | | (n=44) | | | (n=17) |
| *Yes* | | | **83.4%** (70.0-91.5%) | | | **87.8%** (75.4-94.4%) | | | **11.8%** (0.0-27.6%) |

***^*^****Non-Fulani/ Grassland ethnic group.*

^^^*Response not recorded for 1 participant (n=49).*

**Table 2. A descriptive summary of infectious disease awareness in pastoralist and dairy farmers from cross-sectional studies.**

|  | North West Region  Pastoralists  (95% CI, n=50) | Vina Division  Pastoralists  (95% CI, n=50) | North West Region  Dairy Farmers  (95% CI, n=46) |
| --- | --- | --- | --- |
| Foot and mouth disease (FMD) | | | |
| Are you aware of a disease called "foot and mouth disease"? | | | |
| *Yes* | **97.9%** (86.6-99.7%) | **96.0%** (85.4-99.0%) | **56.5%** (42.0-71.0%) |
| Have any of the cattle presented been sick from FMD? | | | |
| *Yes* | **60.1%** (46.6-72.2%) | **76.4%** (62.6-86.2%) | **7.7%** (0.0-18.1%) |
| Have any of your cattle died from FMD? | | | |
| *Yes* | **32.9%** (21.5-46.8%) | **16.8%** (8.8-29.7%) | **3.8%** (0.0-11.4%) |
| Fasciolosis | | | |
| Are you aware of a disease called "fasciolosis"? | | | |
| *Yes* | **80.1%** (68.1-88.3%) | **89.9%** (78.4-95.6%) | **21.7%** (9.7-33.8%) |
| Have any of the cattle presented been sick from fasciolosis? | | | |
| *Yes* | **47.5%** (32.3-63.3%) | **34.4%** (22.4-48.9%) | **0.0%** (0.0-7.7%) |
| Have any of your cattle died from fasciolosis? | | | |
| *Yes* | **35.2%** (21.6-51.8%) | **10.7%** (4.5-23.4%) | **0.0%** (0.0-7.7%) |
| Have you been informed of any cattle sold or slaughtered have fasciolosis? | | | |
| *Yes* | **15.2%** (6.9-30.1%) | **28.3%** (17.1-43.0%) | **0.0%** (0.0-7.7%) |
| Bovine tuberculosis (bTB) | | | |
| Are you aware of a disease called "bovine tuberculosis"? | | | |
| *Yes* | **76.6%** (63.4-86.1%) | **40.8%** (30.1-52.5%) | **73.9%** (61.1-86.7%) |
| Have any of the cattle presented been sick from bTB? | | | |
| *Yes* | **60.4%** (43.8-75.0%) | **43.2%** (24.9-63.6%) | **2.9%** (0.0-8.7%) |
| Have any of your cattle died from bTB? | | | |
| *Yes* | **23.5%** (13.1-38.5%) | **8.5%** (2.1-27.8%) | **0.0%** (0.0-7.7%) |
| Have you been informed of any cattle sold or slaughtered have bTB? | | | |
| *Yes* | **23.5%** (13.1-38.5%) | **8.5%** (2.1-27.8%) | **0.0%** (0.0-7.7%) |
| Had a positive SCITT (1+ animal) in their herd | | | |
| *Yes* | **NA** | **NA** | **4.3%** (0.0-10.3%) |
| Zoonotic disease | | | |
| Do you know any diseases which people can get from consuming cow's milk? | | | |
| *Yes* | **28.3%** (17.3-42.6%) | **26.1%** (15.6-40.3%) | **56.5%** (42.0-71.0%) |
| Cattle keeper named zoonotic tuberculosis as a disease transmitted from consuming cow's milk. | | | |
|  | **9.7%** (4.1-21.1%) | **2.0%** (0.3-12.3%) | **21.7%** (9.7-33.8%) |

**Table 3. A descriptive summary of pastoralist and dairy farmer’s dairy practices cattle from cross-sectional studies.**

|  | North West Region Pastoralists  (95% CI, n) | Vina Division Pastoralists  (95% CI, n) | North West Region Dairy Farmers  (95% CI, n) |
| --- | --- | --- | --- |
| Family members | | | |
| Milk consumed by family | **87.7%**  (77.6-93.6%, 50) | **96.0%**  (85.4-99.0%, 50) | **87.0%**  (77.1-96.8%, 46) |
| Milk processed for family | **74.0%**  (61.7-83.4%, 44) | **95.8%**  (84.8-98.9%, 48) | **100%**  (91.2-100%, 40) |
| Milk soured for family | **55.4%**  (43.8-66.5%, 44) | **87.1%**  (75.0-93.8%, 48) | **0.0%**  (0.0-8.8%, 40) |
| Milk heated for family | **60.8%**  (47.1-73.0%, 44) | **87.5%**  (75.5-94.2%, 48) | **100%**  (91.2-100%, 40) |
| Non-family members | | | |
| Milk consumed by non-family members | **42.2%**  (29.4-56.1%, 50) | **16.5%**  (10.0-26.1%, 50) | **87.0%**  (77.1-96.8%, 46) |
| Milk processed for non-family members | **46.9%**  (27.6-67.1%, 21) | **9.0%**  (1.3-41.0%, 9) | **27.5%**  (13.5-41.5%, 40) |
| Milk soured for non-family members | **41.8%**  (23.5-62.7%, 21) | **9.0%**  (1.3-41.0%, 9) | **0.0%**  (0.0-8.8%, 40) |
| Milk heated for non-family members | **33.0%**  (17.0-54.2%, 21) | **9.0%**  (1.3-41.0%, 9) | **27.5%**  (13.5-41.5%) |
| Dairy products produced | | | |
| Yoghurt | **61.4%**  (50.0-71.7%, 42) | **83.6%**  (70.8-91.4%, 33) | **45.7%**  (31.1-60.2%, 22) |
| Butter | **65.7%**  (52.7-76.7%, 42) | **75.4%**  (62.6-84.9%, 33) | **8.7%**  (0.5-16.9%, 22) |
| Cheese | **0.0%**  (0.0-8.4%, 42) | **0.0%**  (0.0-10.6%, 33) | **2.2%**  (0.0-6.4%, 22) |
